# Going beyond efficacy: strategies for cancer pain management

**DOI:** 10.3747/co.v15i0.201

**Published:** 2008-01

**Authors:** J. Myers, N. Shetty

**Affiliations:** * Palliative Care Consult Team, Sunnybrook Health Sciences Centre, and Department of Family and Community Medicine, University of Toronto, Toronto, ON; † Department of Family and Community Medicine, University of Toronto, Toronto, ON

**Keywords:** Cancer pain management, palliative care

## Abstract

Despite great advances in the fields of pain management and palliative care, pain directly or indirectly associated with a cancer diagnosis remains significantly undertreated. The present paper reviews the current standard for cancer pain management and highlights new treatments and targeted interventional techniques.

## 1. INTRODUCTION

At least 70% of cancer patients will experience pain at some point during the course of their illness 1,2. Pain may arise directly from the underlying oncologic condition, and it may also result from the therapy intended to modify the disease. The knowledge and skill required to address cancer pain has evolved to the point that effective pain management is considered by many to be a fundamental human right and that failure to provide effective pain management is considered to be poor medicine and unethical 3.

Although opioids remain the primary class of medications indicated in the treatment of chronic cancer pain, greater attention has been given to the utility of adjuvant analgesics. Different types of pain vary in the extent to which they respond to various classes of medications, suggesting the need for a clear understanding of pain pathophysiology and an individualized management strategy. The present paper reviews the basics of cancer pain management and addresses some of the more complex clinical pain syndromes, focusing on the medical and interventional therapies currently available.

## 2. REVIEW OF THE BASICS IN CANCER PAIN MANAGEMENT

Pain in patients with cancer is a symptom that can be well managed, and yet it is a consistently undertreated problem 2,3. Physician factors that have previously been reported to hinder treatment of pain include “difficulty with assessment” and “a lack of knowledge about the many tools available to relieve this symptom” 2,3.

The crucial initial step in effective pain management is patient identification through inquiry and observation. Identification can be achieved by universal implementation of valid screening tools. Follow-up steps must include a detailed pain assessment and accurate classification, which together will allow the clinician to select the combination of therapies most likely to provide relief.

### 2.1 Types of Cancer-Related Pain

Cancer-related pain can be classified into two main types, nociceptive and neuropathic:

- **Nociceptive pain** occurs when tissues surrounding nociceptive fibres are injured or stressed. Nociceptive pain can be further divided into somatic pain (which originates in the skin, bones, joints, or muscles) and visceral pain (which originates in internal organs) 1. Patients with somatic pain are usually able to localize the pain well. They will often report an ache that worsens with movement of, or pressure on, the affected area. Visceral pain, usually described as a cramping or pressure-like pain, is often not well localized 1.
- **Neuropathic pain** is pain related to damage or dysfunction of the nervous system, and this pain type is described in more detail in its own subsection, later in this article.

Patients may simultaneously experience more than one type of pain at more than one location, with each pain being likely to respond in varying degrees to various therapies.

### 2.2 The Analgesic Ladder

In an attempt to simplify pain management for clinicians, the World Health Organization devised a medication algorithm known as the “3-step analgesic ladder” 4. In brief, if medications are required to treat mild cancer pain, non-opioids (acetaminophen, acetylsalicylic acid) should first be introduced. If pain persists, or if at presentation it is moderate to severe, opioids should be introduced. Initially, “weak opioids” (codeine, tramadol) should be prescribed; if maximum doses are reached, the weak opioids should be rotated to “strong opioids.” The strong opioids include morphine, oxycodone, hydromorphone, fentanyl, and methadone.

On their own, the strong opioids have no maximum dose. But it is important to note that, although oxycodone is a strong opioid, dosing for combination products containing both short-acting oxycodone and acetaminophen is limited by the maximum allowable daily dose of acetaminophen. Such combination agents are therefore considered appropriate for step 2 of the analgesic ladder.

Although meperidine is considered a strong opioid, it is not used in the cancer pain setting, because consistent use leads to the accumulation of normeperidine in the body and a lowering of the seizure threshold 5.

Table I outlines practical suggestions for initiating opioids.

### 2.3 General Dosing Considerations for Analgesics

If around-the-clock dosing of short-acting opioids is required, the patient should receive a dose every 4 hours 6. Most short-acting opioids have a half-life of 3–4 hours, and for oral preparations, the maximum concentration is reached within 60 minutes (30 minutes by the subcutaneous route, 15 minutes by the intravenous route) 6,7.

“Breakthrough pain” refers to pain experienced despite around-the-clock analgesia. Given the time required to reach maximum concentration, a breakthrough dose should be available to patients once every hour. When initiating an opioid, calculate the breakthrough dose to be at least half of the routine 4-hourly dose, or 10%–20% of the total daily dose of opioid 2.

Long-acting or extended-release opioids have a half-life of approximately 12 hours; most patients should therefore receive them twice daily 8. Long-acting formulations should not be used on an as-needed basis because of their delayed onset of action.

In general, patients should receive the same opioid for routine and breakthrough dosing. The exception is fentanyl, because a breakthrough form is not currently available in Canada. Although on its own fentanyl is a short-acting opioid, it is commonly used in the form of a transdermal patch 6. Patches form a depot under the skin, which slowly releases fentanyl into the subcutaneous tissue and thence into the bloodstream. Pain relief from a fentanyl patch begins 8–12 hours after application, and the patch needs to be changed once every 72 hours 6. A recent report from the Institute for Safe Medication Practices reminded clinicians that fentanyl patches should not be used in the setting of acute pain or for patients who are opioid-naïve 9.

Table II provides an overview of equianalgesic dosing for cases in which opioid rotation is required. There is no consistency in reported ratios, and despite oversimplification, sufficient evidence exists to support the numbers used in the table 2,5,6. Because of a nonlinear ratio with other opioids, fentanyl is not included.

If pain begins to stabilize on a routine dose of a short-acting opioid, the long-acting equivalent of the same opioid should be substituted. The availability of hourly breakthrough dosing should not change.

Whenever possible, opioid side effects should be prevented 10–12. Table III summarizes common side effects and suggests management strategies. Notably, nausea and sedation often resolve within 3–5 days, but ongoing prevention of constipation essential. Stool softeners, stimulant laxatives, and if needed, osmotic laxatives should be prescribed with every opioid initiation.

## 3. AGENTS FOR SPECIAL TYPES OF CANCER PAIN

### 3.1 Bony Pain

The effective management of cancer pain related to primary or metastatic bony disease may include classes of analgesic drugs other than opioids—for example, acetaminophen and nonsteroidal anti-inflammatory drugs (nsaids) 13. The use of nsaids tends to be limited by side effects and concerns about gastrointestinal and renal toxicity. The utility of these drugs initially improved with the advent of the cyclooxygenase-2 selective inhibitors, which lack significant gastrointestinal and renal toxicity, but recent associations with heart disease may affect availability 14,15.

Strong evidence exists for the use of bisphosphonates to reduce metastatic bone pain associated with lung, prostate, and renal cancers 16. The more potent bisphosphonates (pamidronate, zoledronate, and ibandronate) elicit more durable responses than clodronate does 16. The optimum dose and duration of treatment are unknown; however, loading doses (particularly of ibandronate) can reduce refractory bone pain within days 16. Side effects are mild, but renal function must be monitored, particularly with zoledronate 13,16.

### 3.2 Neuropathic Pain

The possible neuropathic component of cancer pain is frequently underdiagnosed or inadequately treated—or both 17. Patients may have great difficulty finding words to describe the sensation, but they may use terms such as “aching,” “burning,” “stabbing,” or “pressure-like.” The description may include a component of “shooting” or “radiating” and the location can be anywhere in the dermatomal region innervated by the damaged neural structure.

Several mechanisms have been proposed to mediate nerve damage or injury expression 18. Peripherally, regeneration after nerve damage can result in the development of neuroma and uncontrolled neuronal firing. This process is thought to be mediated mainly through increased expression of both sodium and voltage-gated calcium channels; hence, these receptors have become the main target of several drugs intended to alter the expression of neuropathic pain. Serotonin and norepinephrine are known to pre-synaptically mediate descending inhibition of ascending pain pathways in the brain and spinal cord, creating a second target for neuropathic analgesics. In addition, heightened sensitivity of spinal neurons is mediated by the *N-*methyl-d-aspartic acid (nmda) receptor, making for a third target.

Although often used as first-line therapy, opioids may have limited efficacy in the management of neuropathic pain 19–21. As a result, improved pain management may be achieved by introducing medications that target one or more of the foregoing pathways.

#### 3.2.1 Anticonvulsants

##### Gabapentin

Gabapentin, first licensed in 1994, was designed as a γ-aminobutyric acid analogue intended to reduce seizure activity 22. Several mechanisms have been postulated to explain the utility of gabapentin in the setting of neuropathic pain. It is known to act centrally at the level of the dorsal horn neurons by binding to calcium channels. It requires 3-times-daily dosing and is excreted unchanged by the kidneys, requiring dosing adjustment in the setting of renal insufficiency 23. Because no enzymatic metabolism occurs in the liver, gabapentin has no significant drug interactions.

No randomized trials have examined the efficacy of gabapentin in the setting of cancer pain. In the management of diabetic neuropathy, gabapentin produces greater pain control with fewer side effects than amitriptyline does 24. The dosing schedule in Table IV addresses both the pharmacodynamics and the potential side effect of somnolence.

##### Pregabalin

Structurally similar to gabapentin, pregabalin was designed to have greater bioavailability and a greater affinity for the same calcium channels blocked by gabapentin 25. The linear pharmacokinetics of pregabalin allow both for twice-daily dosing and for more rapid titration than are seen with gabapentin 26. Clinical efficacy is apparent within 1 week, and with gabapentin, pregabalin must be dose-adjusted in the setting of renal insufficiency 26. Starting doses tend to be 75 mg twice daily, with titration to a maximum total daily dose of 600 mg.

In the setting of cancer pain management, clinical efficacy comparisons have not been made between gabapentin and pregabalin. If either gabapentin or pregabalin must be discontinued, the drug should be tapered over a 1- to 3-week period to prevent withdrawal syndrome (symptoms of which include nausea, headache, diarrhea).

Clinicians must educate themselves regarding medication reimbursement issues, because many drug benefit plans will not cover gabapentin or pregabalin for neuropathic pain unless documentation of poor response to less-expensive medications is provided.

##### Other anticonvulsants

Agents including carbamazepine, phenytoin, valproate, and clonazepam are occasionally used in the setting of cancer pain, but evidence for their benefit is limited 27. Potential side effects and drug interactions often limit the clinical utility of these agents, but they should be considered for complex neuropathic pain syndromes.

#### 3.2.2 Antidepressants

First reported to reduce neuropathic pain in 1977, tricyclic antidepressants (tcas) are commonly used in the setting of cancer pain 28. No single tca has been found to have greater efficacy than any other for neuropathic pain; side effects tend to be the limiting factor in titrating. Nortriptyline tends to have the least anticholinergic properties, and the initiating dose should be 10–25 mg once daily, taken at night.

The newer selective serotonin (and serotonin–norepinephrine) reuptake inhibitors, have been studied in the setting of diabetic neuropathy and trigeminal neuralgia. There is moderate evidence that these medications can provide can provide relief from neuropathic pain; however, tcas remain the antidepressant of choice 29,30.

#### 3.2.3 Corticosteroids

Corticosteroids possess analgesic properties for several cancer pain syndromes, including both neuropathic and bony pain 31. The risk of adverse effects increases with dose and duration of therapy alike; however, these medications may contribute to substantial relief in the setting of acute pain. Corticosteroids should be tapered when implementing the long-term management plan.

#### 3.2.3 Methadone

Developed in Germany during World War ii, methadone was initially designed for use in pain management; however, for several decades, this synthetic opioid was used preferentially in the clinical setting of opioid addiction 32. Recently, though, with improved understanding of methadone’s complex pharmacodynamic and pharmacokinetic properties, the compound is rapidly becoming an essential tool in the management of complex cancer pain.

Methadone exists as a racemic compound: one isomer is a μ-receptor agonist; the other is responsible for inhibition of the nmda receptor and the pre-synaptic reuptake of both norepinephrine and serotonin 33. For patients with a neuropathic component to their cancer, methadone may have a significant role.

Methadone’s rapid absorption is followed by tissue distribution and a highly variable elimination phase intended to maintain plasma levels. The resulting half-life varies greatly—from 4 hours to 120 hours—among individuals 33. Because of this variability (and even in the absence of dose adjustment), the possibility of unexpected toxicity exists for several days after dosing is initiated.

Methadone is metabolized in the liver via the cytochrome P450 system, specifically the CYP3A4 group of enzymes 33. Although not a comprehensive list, Table V highlights some of the important drug interactions.

Methadone has no active metabolites, and no adjustments in dosing need be made in the setting of renal insufficiency. The same formulation of methadone is used for both routine and breakthrough dosing. Because of methadone’s complex pharmacodynamics and pharmacokinetics, breakthrough dosing differs from that of other opioids, tending to be offered every 3 hours at maximum. The onset of analgesia with methadone is 20–30 minutes for oral preparations, and routes of administration include rectal, subcutaneous, intravenous, epidural, and intrathecal 33.

Several models for rotating to methadone from another opioid have been devised 34–36. The equianalgesic ratio and subsequent methadone dosing regimen depend on both the duration of exposure and the dose of the patient’s current opioid. The complexity involved in prescribing methadone for pain management in Canada means that both a specialized federal license and an identified mentor are required.

Methadone is available at very low cost, which makes it an attractive medication for pain management in developing countries. One study compared methadone with morphine as a first-line opioid agent and found equal efficacy in the setting of cancer pain for opioid-naïve patients 37.

##### Case study

Mike, a 33-year-old man with recurrent metastatic rectal carcinoma presents with severe burning rectal pain. He is unable to sit for more than 30 minutes at a time despite titration of both his long-acting hydromorphone to 84 mg three times daily and gabapentin to 1200 mg three times daily. Opioid rotation to methadone proved somewhat beneficial, because at a dose of 50 mg three times daily, Mike was able to cut back his breakthrough requirement during the day to four 15-mg doses of methadone. He was referred to interventional anesthesia for an impar ganglion block.

#### 3.2.4 Interventional Cancer Pain Management

Despite use of the original World Health Organization three-step analgesic ladder for cancer pain management (that is, appropriate oral opioid use), up to 25% of people with cancer may continue to experience pain 2,38. In 1996, a fourth step, “invasive therapy” was added to the guidelines 39. Invasive therapy (“interventions”) should be considered for patients whose pain is not responding to escalating doses of opioids and adequate adjuvant medication, whose pain is likely to be opioid-insensitive (that is, has a neuropathic component or is secondary to bony metastases, and so on), or in whom analgesia is producing intolerable side effects 40. Because of the complexity of the pain syndromes requiring interventions, the interventional procedures should be thought of as adjuvants to standard analgesic regimens. If resources permit, they should be used as soon as the necessity becomes clear.

Neurolysis attempts to interrupt the neural pathway thought to be responsible for the pain syndrome. This interruption can be achieved through anesthesia, including chemical (injection of agents), thermal (cryoablation and radiofrequency), and surgical (cordotomy) techniques 40. The sympathetic chain runs along the vertebral column and makes a good target for intervention at the level determined to be appropriate for the specific pain complaint. Table VI outlines the five main sites for potential block, with their associated pain syndromes.

Continuous epidural, subarachnoid, or intrathecal infusion of an opioid and adjuvants as required is now routinely considered in the management of patients with refractory cancer pain 40. The usual indication is pain in the lower half of the body that cannot be managed at opioid doses below those associated with intolerable and unmanageable somnolence or cognitive impairment 41. The addition of a local anesthetic or other drug to the opioid may provide significant analgesia when intraspinal opioids alone are insufficient 42. The limiting factors tend to be inpatient or outpatient resources.

##### Case study

Jim, a 45-year-old man with metastatic renal cell carcinoma to the pelvis, describes severe, deep, stabbing pain in the right buttock that radiates down the right leg. Trials of several opioids in combination with neuropathic adjuvants have not brought his pain below a 5 on a pain scale of 10. Sativex (GW Pharmaceuticals, Salisbury, U.K.) and oral ketamine were added to his regimen of methadone and pregabalin, and he is being considered for an intraspinal technique.

#### 3.2.5 Cannabinoids

In Canada, the first buccal cannabinoid spray (Sativex) was recently approved for use in cancer pain. The cannabinoid CB1 receptor acts on pathways that partly overlap with those affected by opioids. It is widely distributed throughout the central and peripheral nervous systems 43. Cannabinoids and opioids may have additive or synergistic analgesic effects because of similarities in the physical distribution of their receptors 44. Cannabinoids have an analgesic effect equal to that of codeine, and in the setting of cancer pain, cannabinoids may have a role in management of a neuropathic component 44–47. Sativex is sprayed into the mouth, under the tongue, or onto the inside of the cheek. The starting dose for an adult is not more than 1 spray every 4 hours, to a maximum of 4 sprays, on the first day. In patients with cancer, the average dose of Sativex is 8 sprays spread evenly throughout the day.

#### 3.2.6 Ketamine

Ketamine has been used with some success to reduce the dose of opioid and to improve pain control in the setting of cancer pain 48–50. It acts on the central nervous system in numerous ways, but its effect as a nmda receptor antagonist is thought to be responsible for its utility in treating opioid-resistant neuropathic pain 51. Trials involving patients with cancer-related pain have been performed with ketamine administered in a variety of dosing schedules and routes (intrathecal, epidural, intravenous, subcutaneous, and oral), but there is uncertainty about the conversion ratios between parenteral and oral preparations. Most trials of ketamine have involved relatively low numbers of subjects, but they have shown dramatic reductions in pain with its use 52.

Adverse effects that have been reported with ketamine include tachycardia, hypertension, raised intracranial pressure, and nausea, but the ones that raise the most concern are the psychotomimetic effects such as hallucinations, confusion, and sedation. In some patients, these effects can be avoided by administering haloperidol or a benzodiazepine as a prophylactic 52. However, until more evidence is available, a sensible precaution would be to use ketamine only in patients that have poor pain control despite escalating opioid doses and attempts at opioid rotation. The safest locale for a ketamine trial is an inpatient setting, in which patients can be monitored for adverse effects and doses can be titrated carefully. Careful titration is necessary because, despite its rapid onset of action, ketamine reaches its peak analgesic effect many hours after administration 50. At a dose of 0.5–4.5 mg/kg, a patient can experience anaesthesia while remaining conscious. Lowering the dose to an hourly 0.1–0.3 mg/kg makes ketamine a useful analgesic 50. Protocols for the initiation of a continuous infusion of ketamine are available 50. Hospitalization and specific monitoring are required, but continuous infusion has been shown to be a successful option for refractory pain in the cancer patient. Oral ketamine may have a role in cancer pain management, but has not been well studied 53.

#### 3.2.7 Tramadol

Tramadol exists as a racemic compound: one isomer is both a weak μ-receptor agonist and a serotonin reuptake inhibitor 54; the other isomer inhibits reuptake of norepinephrine. The M1 product of metabolism has six times tramadol’s affinity for the μ-receptor 54. These pharmacokinetic and pharmacodynamic properties suggest that tramadol may have a significant role in the management of cancer pain in the patient describing a strong neuropathic component.

Tramadol has few drug interactions; a notable exception occurs with antidepressants. Caution must be used if a combination of tramadol and either tcas or selective serotonin reuptake inhibitors is being considered; patients receiving combinations of this kind are at great risk of seizure 54. Tramadol should not be used in the setting of advanced renal insufficiency (creatinine clearance below 30 mL/min) or advanced cirrhosis.

Tramadol is available in an extended-release form that is given once daily. Maximum doses of tramadol should not exceed 600 mg daily, and for opioid-naïve individuals, the typical starting total daily dose is 150 mg. The side effects of tramadol are similar to those of other opioids (nausea, sedation, dizziness), with the exception of constipation, which has been found to be significantly less with tramadol than with other opioids 54.

In head-to-head trials, tramadol at high doses has been found to have efficacy equivalent to that of low-dose morphine. The equianalgesic ratio between tramadol and morphine has not been consistently described and falls into the range 4:1–15:1 in the literature 55–57 One head-to-head trial that also included buprenorphine demonstrated equivalent efficacy between the two agents; however, tramadol demonstrated a more tolerable side effect profile 58.

#### 3.2.8 Buprenorphine

Buprenorphine, a synthetic opioid, was developed in the 1960s as a partial μ-receptor agonist 59. Its oral bioavailability is poor, but after development of an associated transdermal delivery system, it was recently reintroduced in cancer pain management. Unlike other transdermal preparations, the polymer matrix system for buprenorphine prevents dose dumping if the integrity of the patch is compromised 60. Clinically, the medication has a ceiling effect at higher doses; this effect is thought to be related to activation of the opioid receptor–like receptor (known to reverse analgesia, producing a counter-opioid response). Buprenorphine is metabolized through the enzyme system that also metabolizes methadone, and it shares the drug interactions that cause concern with methadone administration. The side effects that patients describe are similar to those seen with other opioids, but as with tramadol, buprenorphine is less associated with constipation 61. Patch strengths include 5, 10, and 20 μg per hour. Buprenorphine’s equianalgesic ratio with morphine has not been well described.

Studies in the cancer pain population are limited, but one head-to-head study versus morphine in chronic pain demonstrated significantly greater efficacy and few side effects with buprenorphine 62.

### 3.3 Treatment-Related Pain Syndromes

Chronic pain syndromes related to treatment (systemic therapy, radiation, surgery) are mostly neuropathic in classification 63. The predisposing factors for chronic neuropathic pain following nerve injury are unknown. Any surgical incision, even a minor one, can induce a neuropathic pain syndrome. Radiation-induced fibrosis can cause peripheral nerve injury. The resulting chronic neuropathic pain usually appears months or years after treatment 64. In contrast to nerve injury related to neoplasm, the pain is generally less prominent and slowly progressive. It is often associated with weakness, sensory disturbances, radiation changes of the skin, and lymphedema. Painful dysesthesias, paresthesias, cramps, and restless legs associated with mild weakness, sensory loss, or autonomic dysfunction may follow treatment with neurotoxic chemotherapy (for example, vincristine, cisplatin, paclitaxel). Although most patients report gradual improvement after therapy is discontinued, some develop a persistent, painful polyneuropathy 63.

The treatment algorithm for post-treatment neuropathic pain syndromes is different from that for pain directly related to neoplastic disease, in that first-line medications tend to be anticonvulsants or antidepressants 65. Patients with this type of pain may best be managed in the chronic pain clinic setting.

## 4. EDUCATION OF PATIENTS, CAREGIVERS, AND CLINICIANS

Appropriate use of medications and provision of education to dispel the fears and myths surrounding opioids are crucial components of a cancer pain management strategy. Among clinicians, fears regarding side effects, misconceptions about addiction, and negative previous experiences have been shown to greatly influence opioid prescribing habits 66. A survey by the physicians of the Eastern Cooperative Oncology Group showed that 30% of respondents would not use “strong analgesics” if a patient was expected to survive for more than 6 months, suggesting several unfounded barriers. When opioids are used properly, fewer than 1% of patients with cancer exhibit behaviours consistent with addiction 11,67. Attention to the prevention and management of opioid side effects is needed, but should not delay response. With respect to management of pain in cancer patients, clinicians must routinely assess for barriers that are easily overcome with reassurance and education.

## 5. SUMMARY

Given current understandings of the pain pathways in the body and the cadre of medications and interventions available that may provide complete relief, patients living with cancer should not unnecessarily live with pain. Clinicians have a duty to manage this symptom with intensity and aggression equal to that applied to the cancer itself. With increased education, it is to be hoped that health care professionals will develop greater comfort in using opioids, will introduce adjuvant analgesics appropriately, and will refer to palliative care colleagues when pain or related symptoms in their patients are not well controlled.

## Figures and Tables

**TABLE I:** Summary of opioid dosing in cancer pain management

Initial dosage of “strong opioid” in opioid-naive patient: Fit patient: Morphine 5 mg orally every 4 hours or equivalent Frail patient: Morphine 2.5 mg orally every 4 hours or equivalent Thereafter, titrate to pain relief or unacceptable side effects. Fentanyl patches should not be prescribed for the opioid-naive patient. (If patients are on “weak opioids”—for example, Tylenol 3, combined acetaminophen–oxycodone—they are not opioid-naïve!)
Dosage of “strong opioid” in patients already on opioids (including “weak opioids”): Determine starting dose of “strong opioid” by using equianalgesic table.
When rotating opioids, reduce the calculated dose of the new opioid by approximately 30% (incomplete cross tolerance).

**TABLE II:** Opioid equianalgesics

Drug	Dose	
	Oral	Subcutaneous
Codeine	100 mg	—
Morphine	10 mg	5 mg
Oxycodone	5 mg	—
Hydromorphone	2 mg	1 mg

**TABLE III:** Common opioid side effects and suggested management strategies

Side effect	Management strategy
Nausea	Haloperidol 0.5 mg as needed
	Prochlorperazine 10 mg as needed
Sedation	Educate and reassure as to transient nature of effect
	For persistent sedation, decrease dose, rotate opioid, or consider stimulant (methylphenidate)
Constipation	Stool softener (docusate), *plus* stimulant laxative (senna) given routinely
Urinary retention	Decrease dose or rotate opioid
Pruritus	Antihistamine
	Consider rotation to synthetic opioid
Opioid toxicity(respiratory depression, a delirium, myoclonus, hyperalgesia, seizure, pinpoint pupils)	Decrease opioid dose
	Consider opioid rotation
	Maximize adjuvant analgesics
	Reserve use of naloxone for diagnostic purposes and in setting of severe toxicity only
	Rule out sepsis, hypercalcemia, or other metabolic disturbances that may have predisposed

a Fear of respiratory depression is a common barrier to aggressive pain managment. Although respiratory depression is a serious side effect, it is quite rare in patients whose opioids have been appropriately titrated. Tolerance with regard to respiratory depression follows downregulation of the μ 2 agonist receptor subtype. Downregulation occurs rapidly when opioid dosing is routine.

**TABLE IV:** Suggested titration of gabapentin in setting of neuropathic pain

Day 1	Initiate 300 mg at half strength for 3 days
Day 4	Increase to 300 mg twice daily for 3 days
Day 7	Increase to 300 mg three times daily
Subsequent days	Continue to titrate based on response to a maximum of 3600 mg daily a

a In setting of normal creatinine clearance.

**TABLE V:** Clinically significant drug–drug interactions with methadone

Clinically significant CYP3A4 inducers (that is, they lower methadone concentration)	Amprenavir, efavirenz, nelfinavir, nevirapine, phenobarbital, phenytoin, rifampin, ritonavir
Possibly clinically significant CYP3A4 inducers	Carbamazepine, chronic ethanol
Clinically significant CYP3A4 inhibitors (that is, they increase methadone concentration)	Benzodiazepines, ciprofloxacin, ethanol, fluconazole
Possibly clinically significant CYP3A4 inhibitors	Cimetidine, fluoxetine, omeprazole, quinidine, paroxetine

**TABLE VI:** Common locations along the sympathetic chain amenable to neurolysis

Sympathetic ganglia	Pain syndrome
Cervicothoracic (stellate) ganglion	Neuropathic pain from head and neck cancers, post-mastectomy pain, superior sulcus syndrome
Celiac plexus	Upper abdominal or back pain associated with cancer of the esophagus, pancreas, liver, or stomach
Lumbar ganglia	Flank pain or lower abdominal pain from urologic cancers
Superior hypogastric plexus	Lower pelvic pain from colon, rectal, or gynecologic cancers
Ganglion impar	Perineal and rectal pain from anal or rectal cancer
